# Therapeutic Effects of α1-Antitrypsin on *Psedumonas aeruginosa* Infection in ENaC Transgenic Mice

**DOI:** 10.1371/journal.pone.0141232

**Published:** 2015-10-28

**Authors:** David P. Nichols, Di Jiang, Carrie Happoldt, Reena Berman, Hong Wei Chu

**Affiliations:** 1 Department of Medicine, National Jewish Health and University of Colorado School of Medicine, Denver, Colorado, United States of America; 2 Department of Pediatrics, National Jewish Health and University of Colorado School of Medicine, Denver, Colorado, United States of America; Louisiana State University, UNITED STATES

## Abstract

Cystic fibrosis (CF) is a genetic disease with many airway pathological features, including aberrant epithelial sodium channel (ENaC) function, persistent *Pseudomonas aeruginosa* (PA) infection and neutrophil-dominant inflammation. PA infection in CF airways is difficult to treat due to antibiotic resistance and other factors. Recently, α1-antitrypsin (A1AT) have been shown to be effective to reduce CF airway PA infection. However, there is a dearth of studies about the mechanisms underlying A1AT’s therapeutic effects. The goal of our study is to provide an animal model of A1AT therapy in CF lungs. ENaC transgenic mice with PA infection were used as a CF-like model. Mice were intratracheally treated with PA or saline (control) in a fibrin plug. Two hours after PA infection, aerosolized A1AT were delivered to mouse lungs once daily. At day 1 and day 3 post PA infection, lung inflammation, PA load as well as host defence protein short palate, lung, and nasal epithelium clone 1 (SPLUNC1) were measured. At day 1 post PA infection when A1AT was delivered once to ENaC transgenic mouse lungs, A1AT did not reduce lung inflammation (e.g., neutrophils) and PA load. However, at day 3 post PA infection when ENaC transgenic mice received three repeated A1AT treatments, a significant decrease in airspace inflammation and PA load was observed. Although A1AT prevented the loss of SPLUNC1 in bronchoalveolar lavage fluid of PA-infected wild-type mice, it did not restore SPLUNC1 levels in ENaC transgenic mice. Our current study has provided a valid and quick A1AT therapeutic model in CF-like lungs that may serve as a platform for future mechanistic studies about how A1AT exerts beneficial effects in human CF patients.

## Introduction

Cystic fibrosis (CF), which is caused by mutation in CF transmembrane conductance regulator (CFTR) [1, 2], affects about 30,000 people in the US. One of the most challenging elements of CF pathophysiology is persistent endobronchial infection with bacteria, particularly *Pseudomonas aeruginosa* (PA). Antibiotic resistance, biofilm growth state, and limited drug concentrations within the airway all work to make PA infection in CF difficult to treat [3, 4]. Thus, new and effective ways to reduce bacterial burden in the CF airway are urgently needed.

α1-antitrypsin (A1AT) exerts multiple functions useful in treating PA airway infection [5, 6]. CF airway disease is characterized by exuberant airway neutrophilic inflammation and high concentrations of free neutrophil elastase (NE). It is widely recognized that A1AT can target NE and clinical studies in CF subjects suggest that A1AT reduces both airway neutrophilia and PA load [7]. However, the mechanisms by which this occurs remain unclear–particularly in regards to the apparent antibacterial effects. In our previous study of non-CF mice [6], we demonstrated that A1AT promotes lung bacterial clearance by preventing NE-mediated degradation of short palate, lung, and nasal epithelium clone 1 (SPLUNC1), an airway epithelial cell host defence protein. However, it is unclear whether or not A1AT reduces bacterial infection in the CF lung, and if so, whether A1AT could increase or maintain SPLUNC1 levels in the lung.

In the current study, we tested the hypothesis that in a model of CF-like mouse lungs, A1AT is effective in inhibiting bacterial (e.g., PA) infection and associated inflammation. Furthermore, we hypothesized that A1AT exerts its therapeutic effect by stabilizing SPLUNC1 levels during bacterial infection in CF lungs.

## Materials and Methods

### Animals

Epithelial sodium channel (ENaC) transgenic (Tg) and littermate control wild-type (WT) mice on the C57BL/6 background were used for the current study. ENaC Tg and WT mice were obtained from Jackson Laboratories (Bar Harbor, Maine, USA) and were bred and housed in our biological resource center at National Jewish Health under pathogen-free conditions, and tested to establish that they were virus and *M*. *pulmonis* free. All the animal procedures were approved by the IACUC at National Jewish Health.

### *Pseudomonas aeruginosa* culture

A CF clinical isolate of mucoid *Pseudomonas aeruginosa* (PA) was used in our current mouse study [8]. For each experiment, bacteria were first streaked onto a LB agar plate and cultured for 18–22 hrs at 37^°^C. An individual colony was then inoculated into LB medium and shaked at 37^°^C to grow the bacteria until 1 x 10^9^ CFUs/ml were achieved as determined by spectrophotometry (optical density at 600 nm = 1.0).

### Mouse fibrin plug model of retained *Pseudomonas aeruginosa* lung infection with α1-antitrypsin treatment

PA (1.5x10^7^ CFUs/mouse) was instilled directly into the airway of wild-type (WT) and ENaC Tg mice using two sequential solutions of thrombin and fibrinogen to form fibrin airway plugs, as we previously described [8, 9]. There are several PA infection models including the agarose bead PA and fibrin PA models. Although none of the models perfectly mimics human CF PA infection, the fibrin PA model has several advantages. These include the ability of the fibrin plug to retain bacterial infection in the lung and mimic CF airway biofilms, and the fact that large amount of fibrin is found in the PA biofilms [8–10]. Sterile saline preparations of thrombin and fibrinogen were instilled using the same method as the negative control for comparison studies. After 2 hrs of PA infection, purified human alpha-1 proteinase inhibitor (A1AT, Grifols Inc., Research Triangle Park, NC, USA) or bovine serum albumin (BSA, as the A1AT control, Sigma-Aldrich, St. Louis, MO, USA) were aerosolized to mice. To deliver A1AT or BSA, mice were placed in a Plexiglas chamber and treated with aerosolized A1AT or BSA (0.5 mg/ml, total volume = 10 ml) for 30 minutes by using an ultrasonic nebulizer (De Vilbiss) at an airflow rate of 8 L/min [6]. After 24 and 72 hrs of PA infection, lung lavage was performed to collect bronchoalveolar lavage **(**BAL) fluid for cell count, and measurement of KC, SPLUNC1 and NE activity. For mice sacrificed at 72 hr post PA infection, A1AT delivery was repeated at 24 hr and 48 hr post PA infection.

### BAL and lung tissue processing

Mouse lungs were lavaged with 1 ml of sterile saline. Cell-free BAL fluid were stored at –80°C for cytokine analysis and Western blot. Cytospins of BAL cells were stained with a Diff-Quick Kit (IMEB INC., San Marcos, CA, USA), and leukocyte differentials were determined as percentage of 500 counted leukocytes. The left lung lobe from infected mice was homogenized in PBS. The homogenates of left lung lobe were then cultured on LB agar plates to quantify PA levels. The apical lobe of the right lung was fixed in 10% formalin, embedded in paraffin, and cut at 5 μm thickness for H&E staining and histological analysis.

### Lung histological analysis

H&E-stained lung sections were evaluated in a blinded fashion under the light microscope using a histopathologic inflammatory scoring system as we described [11]. A final score per mouse (both infected and uninfected) on a scale of 0 to 26 (least to most severe) was obtained based on an assessment of the quantity and quality of peribronchiolar and peribronchial inflammatory infiltrates, luminal exudates, perivascular infiltrates, and parenchymal pneumonia.

### Western blot analysis of mouse SPLUNC1 protein in BAL fluid

Western blot analysis was carried out to quantify SPLUNC1 protein. In brief, 30 μl BAL fluid was electrophoresed on 10% SDS-polyacrylamide gel electrophoresis, transferred onto a nitrocellulose membrane, blocked with the Western blocking buffer, and incubated with a sheep anti-mouse SPLUNC1 antibody (R&D Systems, Inc., Minneapolis, MN) overnight at 4°C. After washes in PBS with 0.1% Tween 20, the membranes were incubated with an anti-sheep IgG conjugated to horseradish peroxidase. Membranes were stained with Ponceau S solution to normalize total protein load by comparing the 68-kDa protein (corresponding to albumin) levels. Densitometry was performed using the NIH Image-J software. The ratio of SPLUNC1/albumin was used to normalize SPLUNC1 protein measurement in BAL fluid.

### ELISA of mouse KC

KC, a homolog of human IL-8, in mouse BAL fluid was determined by using a mouse KC DuoSet ELISA Development kit (R&D Systems, Minneapolis, MN) as per manufacturer’s instruction.

### Neutrophil elastase (NE) activity assay in mouse BAL fluid

NE activity was detected in mouse cell-free BAL fluid by using the substrate N-(methoxysuccinyl)-Ala-Ala-Pro-Val p-nitroanilide (Sigma-Aldrich) as we previously described [12]. OD values were used to indicate NE activity levels.

### Statistical analysis

Data are presented as means ± SEM. One-way analysis of variance (ANOVA) was used for multiple comparisons, and a Tukey’s post hoc test was applied to illustrate the significant differences between two groups. Student’s *t* test was used when only two groups were compared. A *p* value < 0.05 was considered significant.

## Results

### Therapeutic effect of A1AT on lung bacterial load and inflammation in ENaC transgenic mice

**Fig 1:**
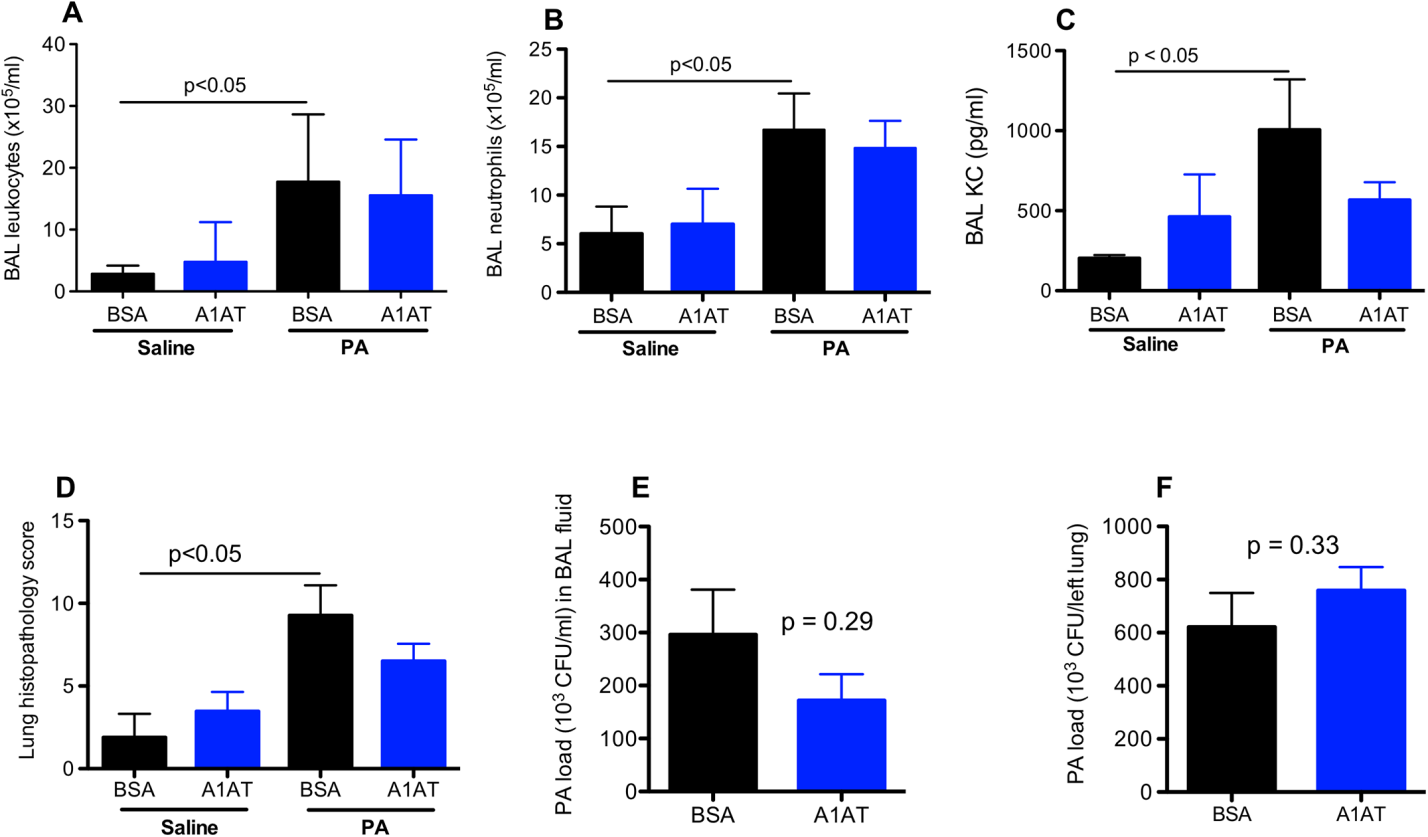
Effects of alpha1 antitrypsin (A1AT) on lung inflammation and *Pseudomonas aeruginosa* (PA) load in ENaC transgenic mice after 1 day of infection. Mice were infected with PA as described in Materials and Methods, and treated at 2 hr post PA infection with A1AT. After 1 day of PA infection, mice (n = 6–8 mice/group) were sacrificed to determine inflammation (A, B, C, D) and PA load (E, F) in bronchoalveolar lavage (BAL) fluid and lung tissue. BSA = bovine serum albumin, serving as a protein control for A1AT. Data are presented as means ± SEM.

**Fig 2:**
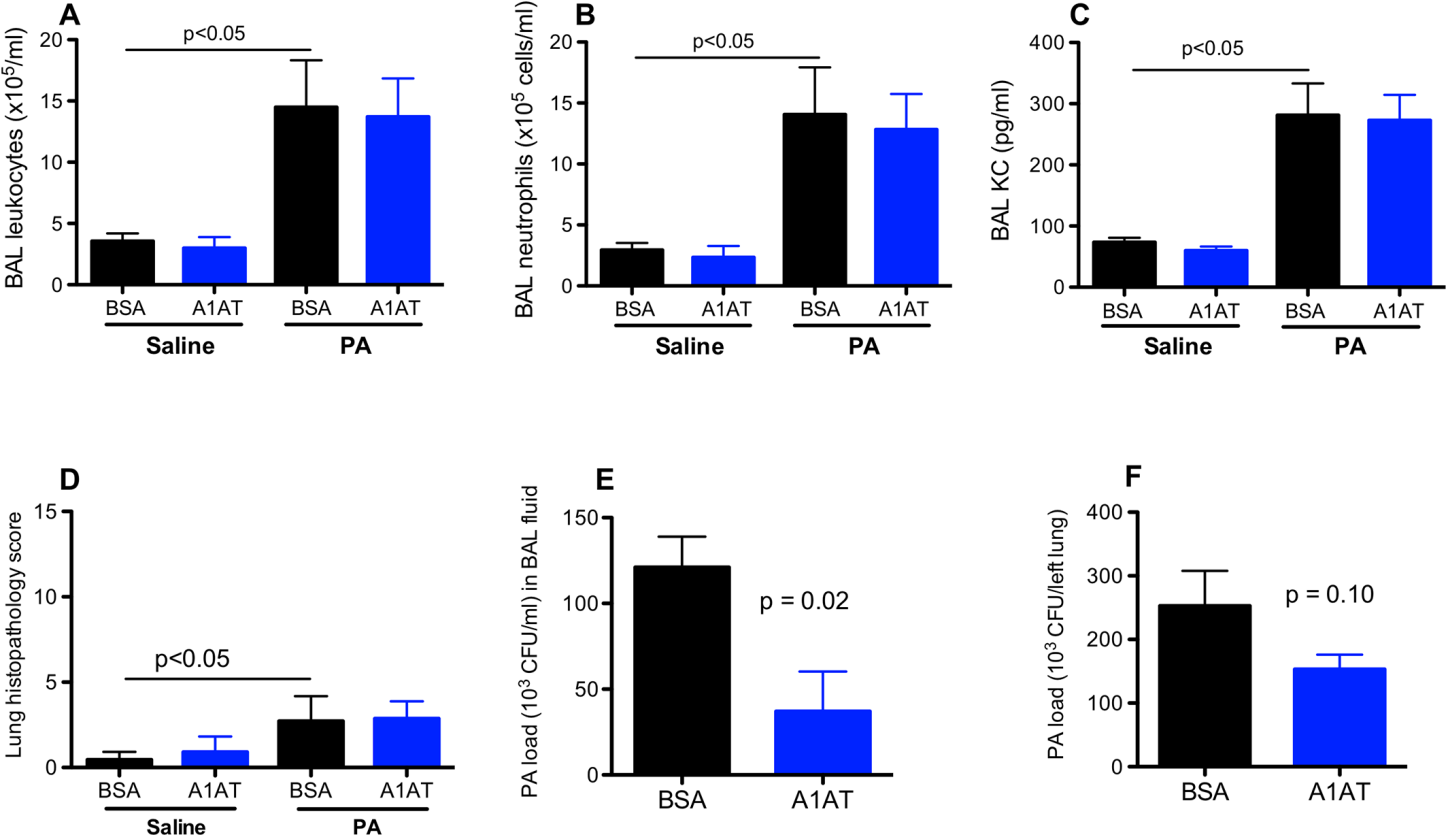
Effects of alpha1 antitrypsin (A1AT) on lung inflammation and *Pseudomonas aeruginosa* (PA) load in wild-type mice after 1 day of infection. Mice were infected with PA as described in Materials and Methods, and treated at 2 h post PA infection with A1AT. After 1 day of PA infection, mice (n = 6 mice/group) were sacrificed to determine inflammation (A, B, C, D) and PA load (E, F) in bronchoalveolar lavage (BAL) fluid and lung tissue. BSA = bovine serum albumin, serving as a protein control for A1AT. Data are presented as means ± SEM.

In the current study, we focused on the therapeutic effect of A1AT in PA-infected ENaC transgenic (Tg) mice. To determine the time course of A1AT’s therapeutic effects, we measured lung inflammation and bacterial load at day 1 and day 3 after PA infection. In ENaC Tg mice infected for 1 day, there was no significant therapeutic effect of A1AT on lung PA load or inflammation as indicated by the levels of leukocytes and KC protein in the BAL fluid as well as lung tissue histopathology scores (Fig 1). In wild-type mice, A1AT also did not reduce the lung inflammation, but moderately decreased the PA load in BAL fluid (Fig 2). A1AT had no observed effect in animals challenged with saline.

**Fig 3:**
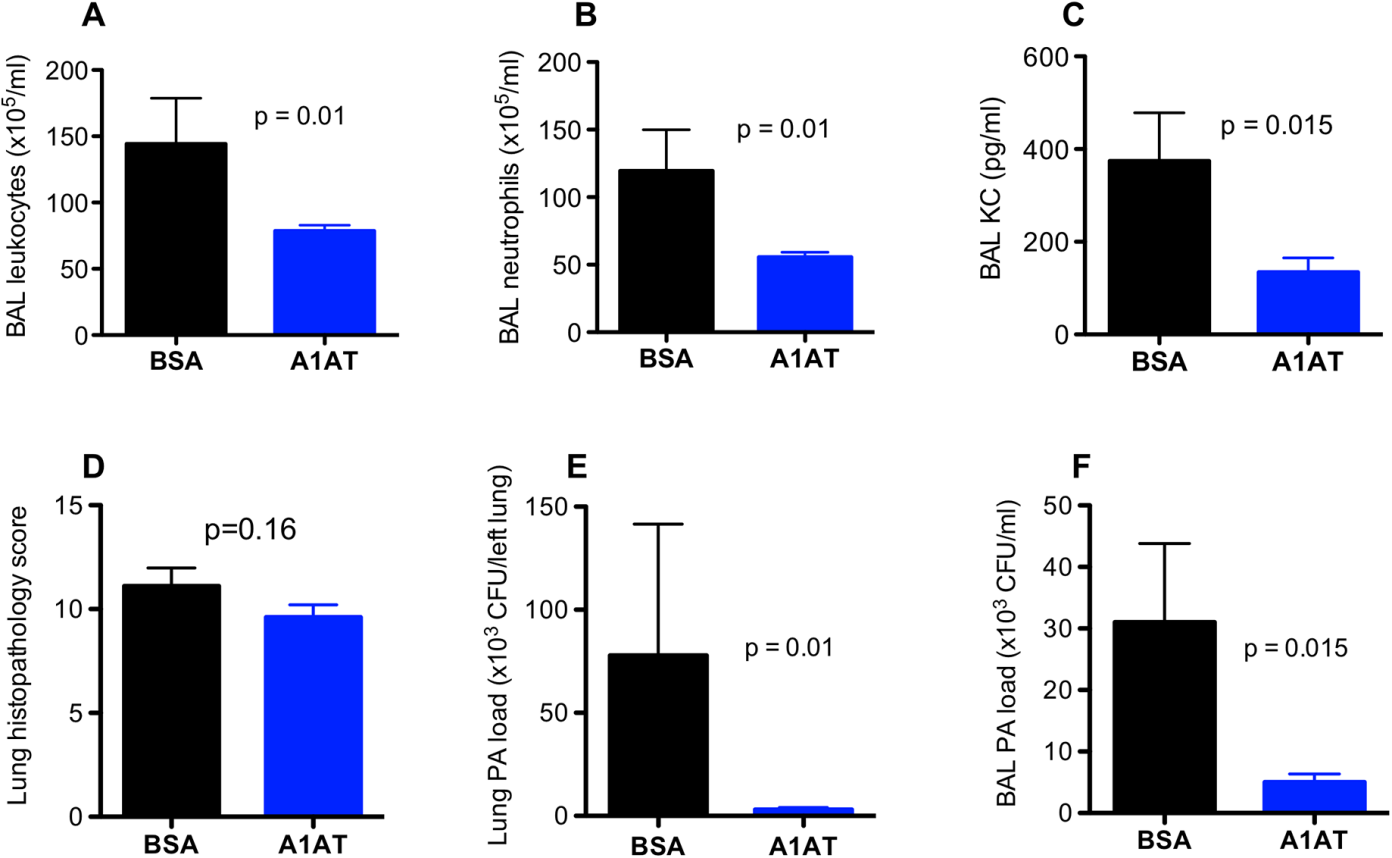
Effects of alpha1 antitrypsin (A1AT) on lung inflammation and *Pseudomonas aeruginosa* (PA) load in ENaC transgenic mice after 3 days of infection. Mice were infected with PA as described in Materials and Methods, and treated at 2 hr, 1 day and 3 day post PA infection with A1AT. After 3 days of PA infection, mice (n = 6–7 mice/group) were sacrificed to determine inflammation (A, B, C, D) and PA load (E, F) in bronchoalveolar lavage (BAL) fluid and lung tissue. BSA = bovine serum albumin, serving as a protein control for A1AT. Data are presented as means ± SEM.

**Fig 4:**
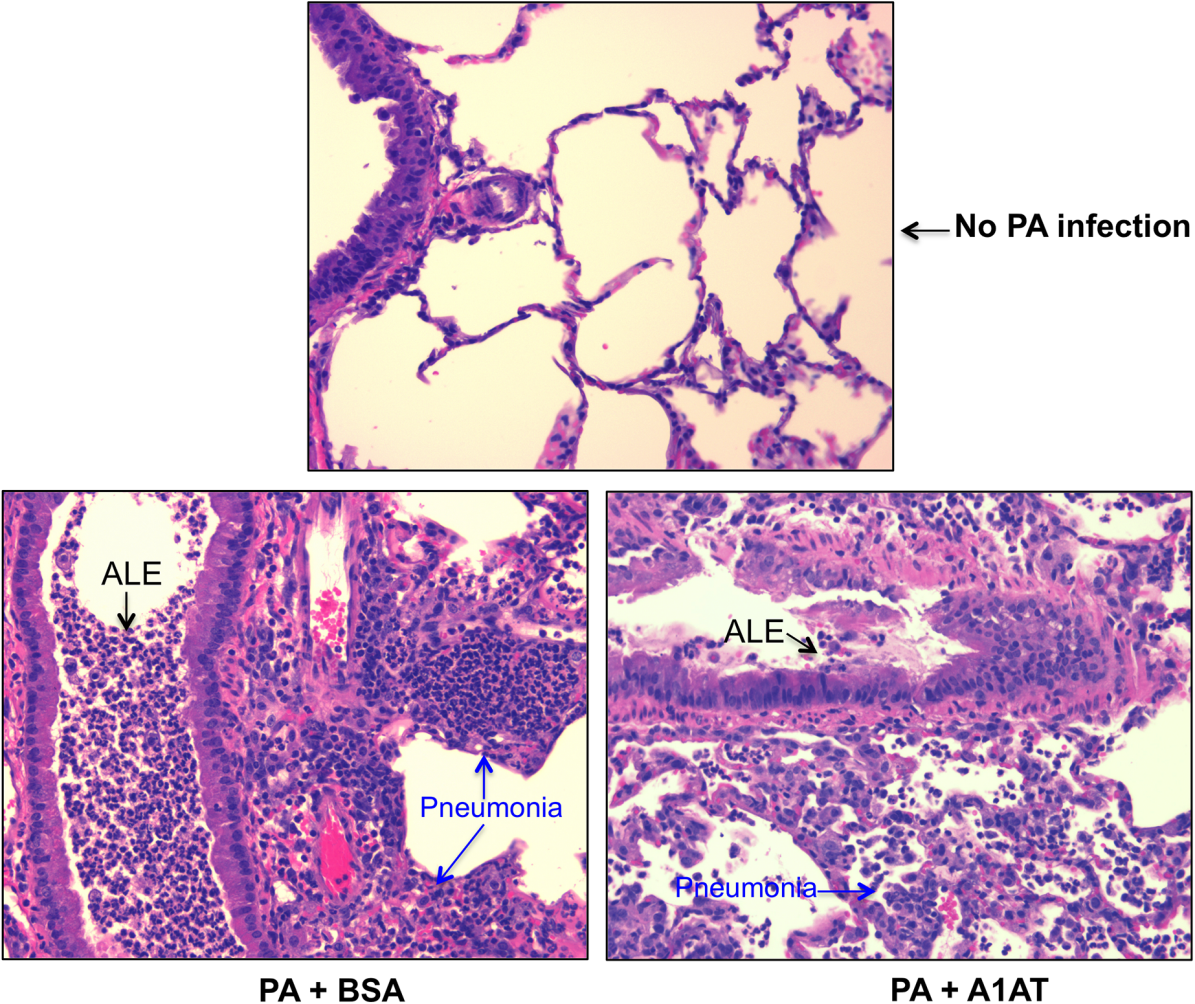
Representative lung tissue histopathology pictures (H&E staining; magnification, x200) from ENaC transgenic mice. Mice were not infected with *Pseudomonas aeruginosa* (PA) or infected with PA in the presence or absence of alpha1 antitrypsin (A1AT) treatment. After 3 days of PA infection, mouse lungs were processed for evaluation of histopathology as described in Materials and Methods. BSA = bovine serum albumin, serving as a protein control for A1AT. ALE = airway luminal exudates.

**Fig 5:**
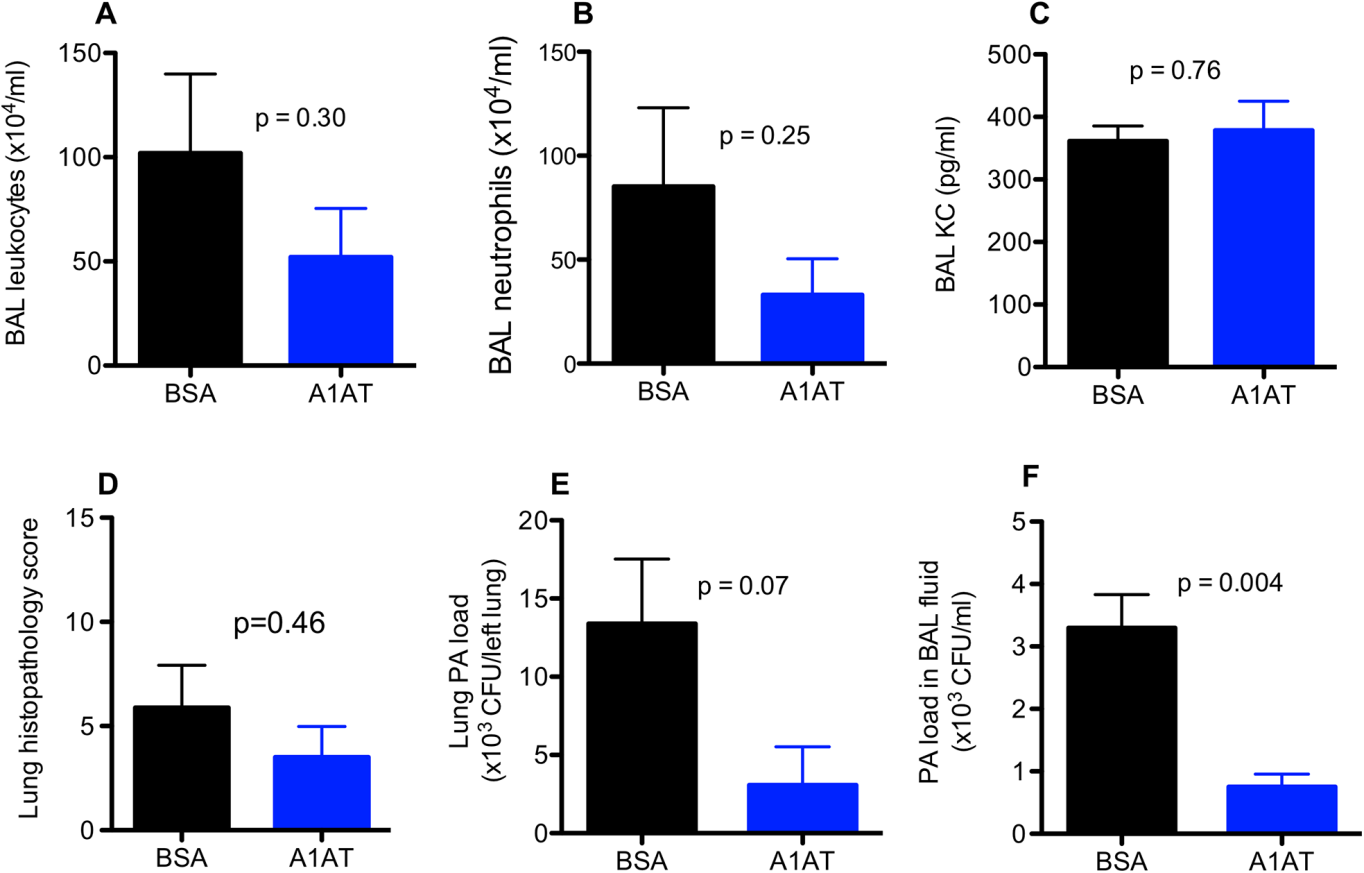
Effects of alpha1 antitrypsin (A1AT) on lung inflammation and *Pseudomonas aeruginosa* (PA) load in wild-type mice after 3 days of infection. Mice were infected with PA as described in Materials and Methods, and treated at 2 hr, 1 day and 3 day post PA infection with A1AT. After 3 days of PA infection, mice (n = 5 mice/group) were sacrificed to determine inflammation (A, B, C, D) and PA load (E, F) load in bronchoalveolar lavage (BAL) fluid and lung tissue. BSA = bovine serum albumin, serving as a protein control for A1AT. Data are presented as means ± SEM.

In contrast to the data on day 1, A1AT treatment in ENaC Tg mice infected with PA for 3 days resulted in significant reduction of PA load in the lung and BAL fluid, as well as leukocytes including neutrophils and KC in BAL fluid (Fig 3). Although lung tissue inflammation as indicated by the histopathology score was reduced by A1AT (Fig 3D), the difference did not reach the level of statistical significance. PA infection induced neutrophil-predominant lung inflammation manifested by pneumonia, peribronchial and perivascular infiltrate of inflammatory cells including neutrophils, lymphocytes and macrophages (Fig 4). We conducted studies to examine the effects of A1AT treatment in PA-infected wild-type mice at day 3 post infection. As in the ENaC Tg mice, A1AT reduced PA load in the lung and BAL fluid (Fig 5), but the decrease of BAL leukocytes including neutrophils in PA-infected wild-type mice did not reach the level of statistical significance. As we did not see any effects of A1AT in saline-treated mice at day 1, we did not treat the ENaC Tg or wild-type mice with saline for our day 3 experiments.

### Neutrophil elastase (NE) activity in lung tissue of A1AT-treated and PA-infected ENaC Tg mice

**Fig 6:**
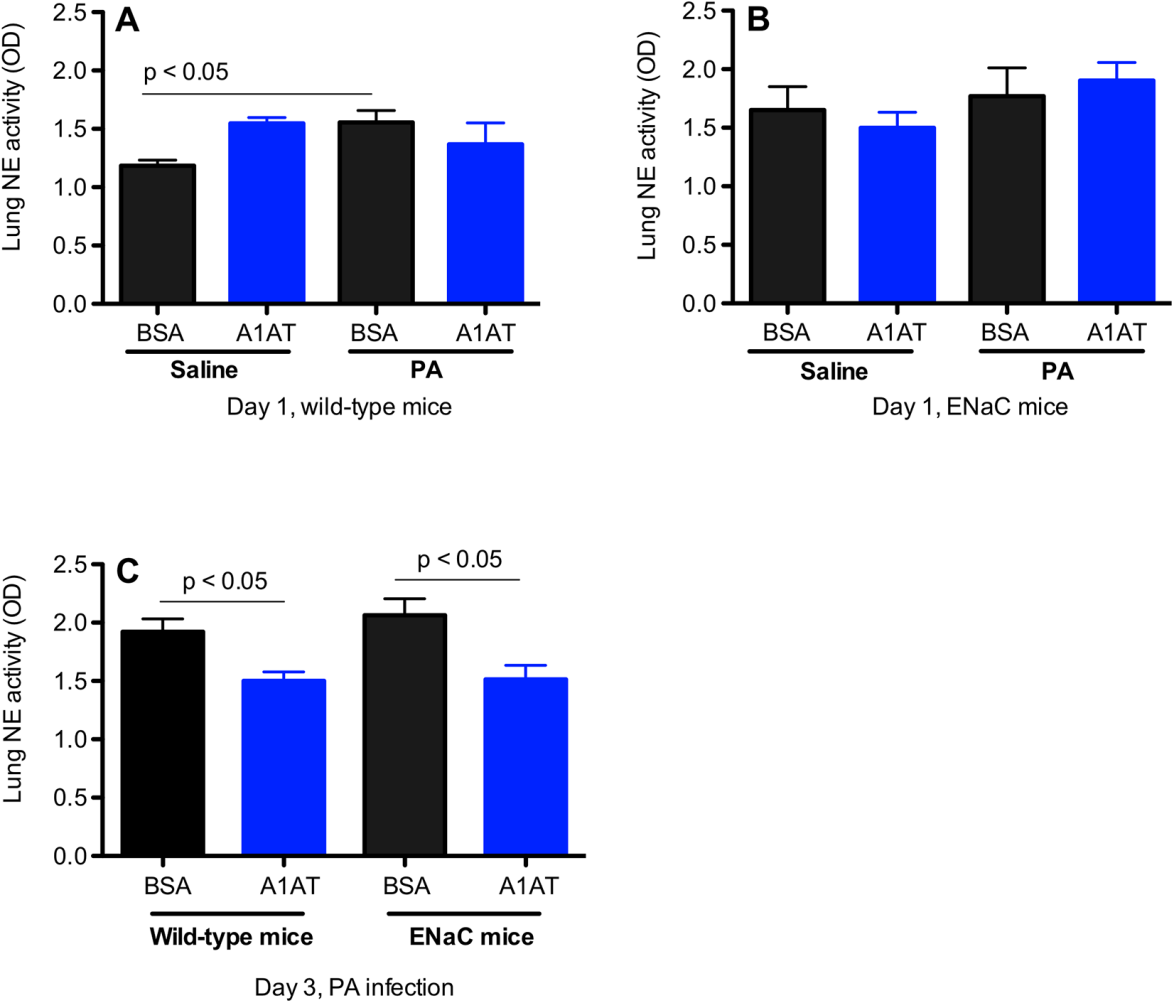
Effects of alpha1 antitrypsin (A1AT) on neutrophil elastase (NE) activity in the lung. Lung tissues from wild-type (A, n = 6 mice/group) and ENaC transgenic mice at day 1 (B, n = 6–8 mice/group) and at day 3 (C, n = 5–7 mice/group) post *Pseudomonas aeruginosa* (PA) infection were collected and homogenized to measure NE activity. BSA = bovine serum albumin, serving as a protein control for A1AT. Data are presented as means ± SEM.

NE activity was measured in lung tissue homogenates to determine if A1AT effectively reduces NE activity in PA-infected ENaC Tg mice. As shown in Fig 6A and 6B, at day 1 post PA infection, although PA increased the lung NE activity, A1AT did not reduce its activity in PA-infected wild-type mice or ENaC Tg mice. However, at day 3 post PA infection, NE activity in the lungs of both wild-type and ENaC Tg mice was significantly inhibited by A1AT treatment. Thus, the NE data are consistent with the therapeutic effect of A1AT on lung PA load and inflammation.

### SPLUNC1 protein in BAL fluid of A1AT-treated and PA-infected CF mice

**Fig 7:**
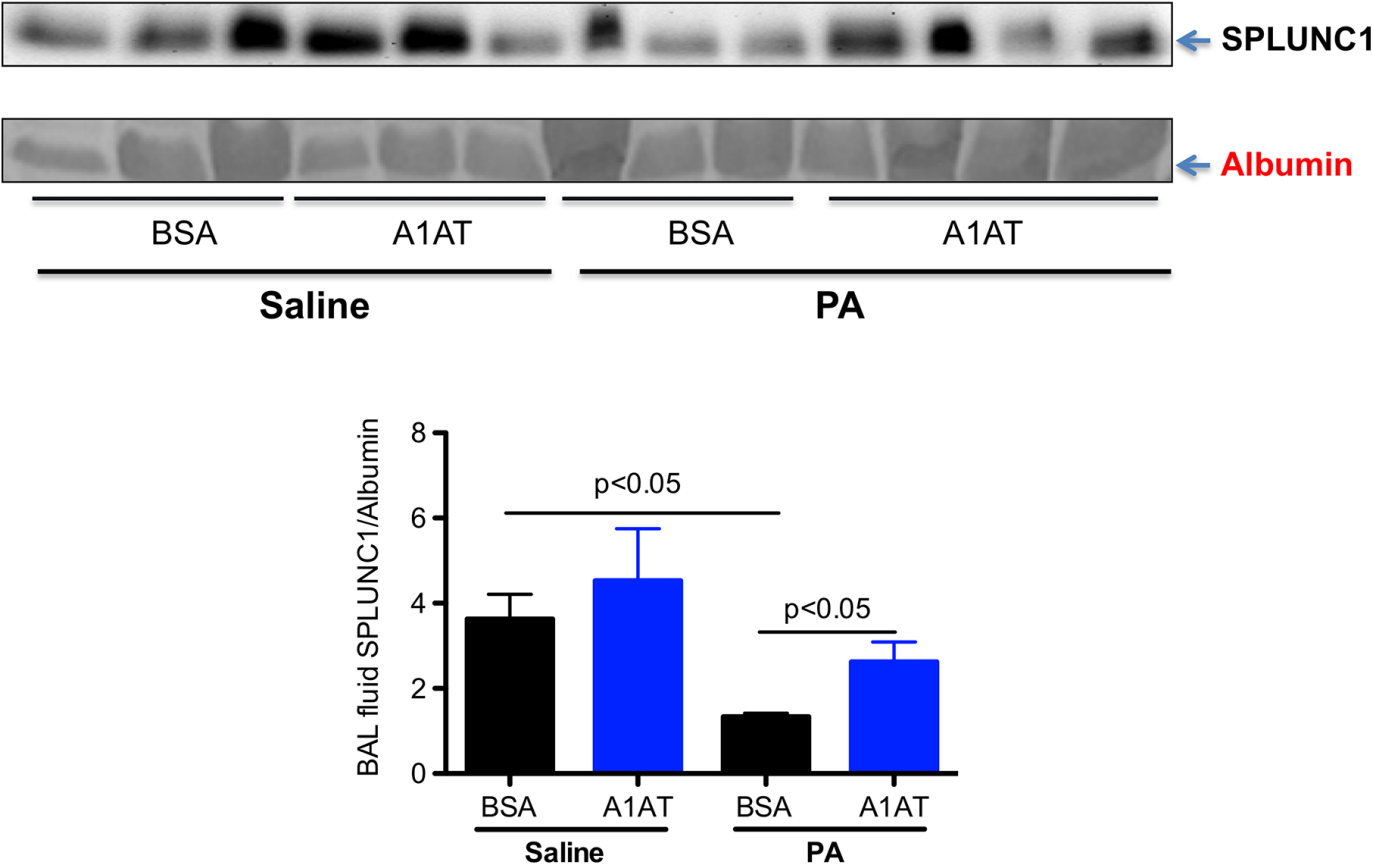
Effects of alpha1 antitrypsin (A1AT) on SPLUNC1 protein levels in bronchoalveolar lavage (BAL) fluid of wild-type mice after 1 day of infection. Mice were infected with *Pseudomonas aeruginosa* (PA) as described in Materials and Methods, and treated at 2 hr post PA infection with A1AT. After 1 day of PA infection, mice (n = 6 mice/group) were sacrificed to determine SPLUNC1 protein levels in BAL fluid by using Western blot. BSA = bovine serum albumin, serving as a protein control for A1AT. Data are presented as means ± SEM.

**Fig 8:**
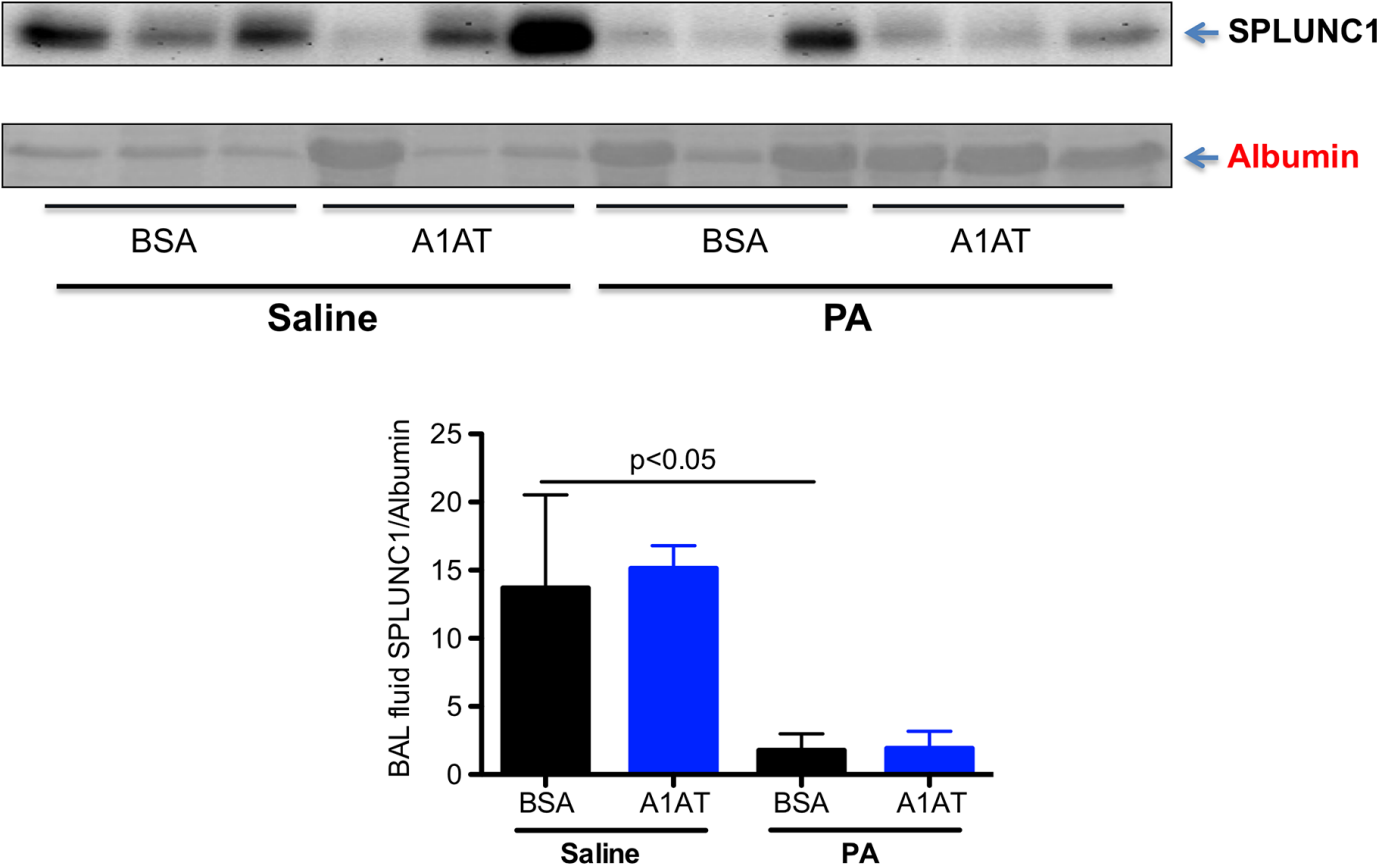
Effects of alpha1 antitrypsin (A1AT) on SPLUNC1 protein levels in bronchoalveolar lavage (BAL) fluid of ENaC transgenic mice after 1 day of infection. Mice were infected with *Pseudomonas aeruginosa* (PA) as described in Materials and Methods, and treated at 2 hr post PA infection with A1AT. After 1 day of PA infection, mice (n = 6–8 mice/group) were sacrificed to determine SPLUNC1 protein levels in BAL fluid by using Western blot. BSA = bovine serum albumin, serving as a protein control for A1AT. Data are presented as means ± SEM.

**Fig 9:**
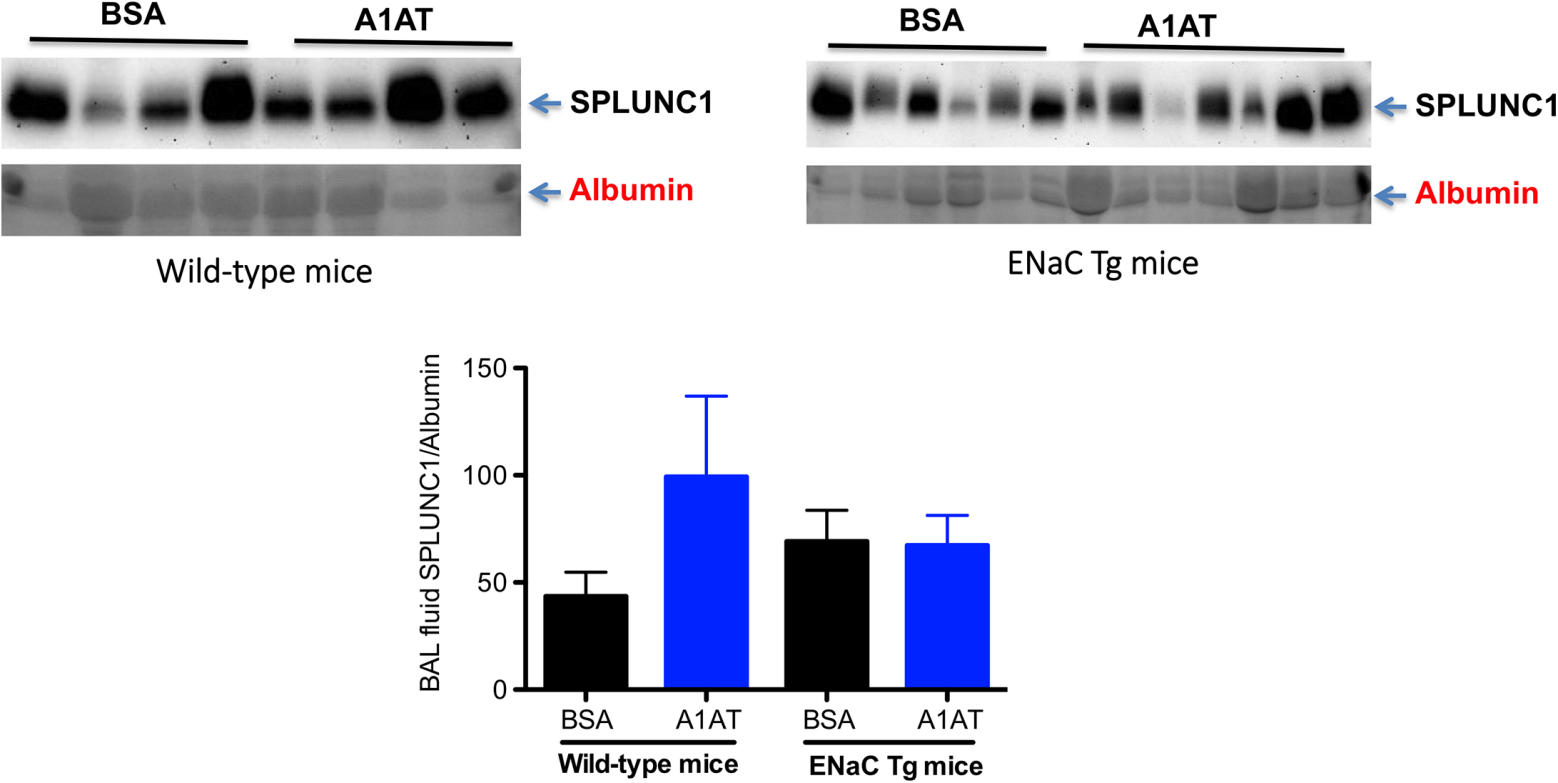
Effects of alpha1 antitrypsin (A1AT) on SPLUNC1 protein levels in bronchoalveolar lavage (BAL) fluid of wild-type and ENaC transgenic (Tg) mice after 3 days of infection. Mice were infected with *Pseudomonas aeruginosa* (PA) as described in Materials and Methods, and treated at 2 hr, 1 day and 3 day post PA infection with A1AT. After 3 days of PA infection, mice (n = 5–7 mice/group) were sacrificed to determine SPLUNC1 protein levels in BAL fluid by using Western blot. BSA = bovine serum albumin, serving as a protein control for A1AT. Data are presented as means ± SEM.

In our previous studies, PA infection in the wild-type mice decreased SPLUNC1 protein levels in BAL fluid after 1 day of infection [6]. Our current study confirmed our previous finding that SPLUNC1 protein levels were significantly lower in PA-infected than in saline-treated wild-type mice, and that A1AT restored the SPLUNC1 levels in PA-infected wild-type mice after 1 day of infection (Fig 7). Although PA infection at day 1 in ENaC Tg mice decreased SPLUNC1 protein levels in BAL fluid, A1AT did not restore or increase SPLUNC1 levels (Fig 8). Similarly, A1AT did not alter SPLUNC1 in BAL fluid of ENaC Tg mice at day 3 post PA infection (Fig 9). In wild-type mice, after 3 days of PA infection, A1AT trended to increase SPLUNC1 levels, but this did not reach the level of statistical significance.

## Discussion

By using a fibrin plug PA airway infection model to prolong the bacterial infection in mice, we observed therapeutic effects of A1AT on both lung inflammation and bacterial load–two major pathological features of CF lung disease. These effects were present following 3 days, but not 1 day, of A1AT airway administration by nebulization.

Although the anti-protease function of A1AT has been well established [13], other potentially beneficial effects of A1AT remain to be determined. Our study in a mouse model of CF-like lung disease with heightened baseline and inducible airway neutrophilic inflammation and repeated A1AT delivery for 3 days is consistent with previous studies [7] in human CF patients that demonstrated the efficacy of inhaled A1AT (25 mg/patient) for 4 weeks in reducing lung inflammation and Pseudomonas load. In the study by Griese M et al [7], A1AT was measured in the induced sputum samples. After 4 weeks of A1AT inhalation, A1AT levels were about 30–35 μg/ml. We did not observe therapeutic effects of a single A1AT treatment after 24 hours of PA airway challenge. This is consistent with the belief that repeated A1AT administration as part of a chronic therapy will be necessary to demonstrate clinically beneficial effects. Our 3-day CF-like mouse model of PA airway infection and A1AT administration reflected the therapeutic benefits of A1AT reported in human CF patients, suggesting that this mouse model may be useful for future studies.

How A1AT inhibits the pro-inflammatory response in PA-infected CF-like lungs is poorly understood. In our recent studies in PA-infected wild-type mice [6], we demonstrated that A1AT significantly reduced lung inflammation and PA load in part through maintaining or even increasing the host defense protein SPLUNC1. In the current study, we found that A1AT increased SPLUNC1 in BAL fluid of wild-type mice, but did not affect SPLUNC1 levels in ENaC Tg mice. The inability of A1AT to preserve SPLUNC1 in PA-infected ENaC Tg mice may be explained by the significantly higher levels of lung inflammation (e.g., 10-fold more neutrophils in BAL and 2-fold higher scores of histopathology on day 3 post PA infection) in these mice than in the wild-type mice. Future studies using higher concentrations or longer administration of inhaled A1AT will be needed to determine if preservation of SPLUNC1 can be achieved. It is also likely that A1AT may have other host defense functions that are not dependent on its rescuing effect on SPLUNC1. For example, surfactant protein A (SP-A) is involved in lung defense against PA [14], but it is also susceptible to degradation by neutrophil elastase [15]. It remains to be determined whether A1AT utilizes other mechanisms such as protection against SP-A degradation and subsequently enhances lung bacterial clearance in ENaC Tg mice. Our previous and current data support that A1AT airway administration decreases both neutrophilic inflammation and bacterial load, and that at least some of this effect is related to inhibition of neutrophil elastase with preservation of SPLUNC1 to help maintain antimicrobial defense. Anti-inflammatory therapy in CF lung disease is often challenged by a concern of increasing susceptibility to the ever-present bacterial infection. Therefore, an immunomodulatory therapy also capable of improving host defense against such pathogens would be a significant addition to currently available CF treatment options.

There are several limitations to the current study. First, we need to better understand the molecular mechanisms by which A1AT inhibits lung inflammation or bacterial load. A recent publication [16] suggests that A1AT suppresses lung inflammation through activating protein phosphatase 2A (PP2A). Whether this occurs in PA-infected ENaC Tg mouse lungs awaits our investigation. Second, CF-like mouse models of PA infection longer than 3 days may provide additional data not present in our study. Unfortunately, attempts to develop CF mouse models of chronic bacterial infection with significant airway neutrophilic inflammation remain challenging and inconsistent. Newer animal models (e.g., ferret, pig) of CF airway disease may be useful in studying the long-term therapeutic effects of A1AT in CF lungs, but face additional challenges. Whether A1AT has a direct antimicrobial effect on PA remains unclear. We conducted preliminary experiments testing A1AT (0.1–5 mg/ml) for 16 hours in a biofilm culture model of PA and did not observe any direct antibacterial effect (data not shown). This suggests that indirect effects of A1AT, including the preservation of SPLUNC1 and other antimicrobial substances, are more likely responsible for reduced airway infection in the context of A1AT inhalation. Lastly, it is not entirely clear why A1AT appears to have a greater anti-inflammatory effect in the airspace (e.g., BAL fluid) than the lung tissue (lung parenchyma, peribronchial and perivascular tissue). We propose that such a differing effect of A1AT on airspace versus lung tissue inflammation may be explained by the heterogeneity of lung inflammation in PA-infected mice, and by the fact that only a small piece of lung tissue was used in the current study for the histopathology evaluation as other pieces of the lung were used for measuring bacterial load and neutrophil elastase activity.

In summary, our mouse CF model of PA infection may serve as an excellent model to investigate the mechanisms of A1AT function *in vivo*, thus overcoming the drawbacks in human CF studies.
